# Constructing Neuroinflammation‐On‐A‐Chip for Traditional Chinese Medicine Extracts Evaluation

**DOI:** 10.1002/smmd.70032

**Published:** 2026-03-19

**Authors:** Xirui Wang, Xiang Lin, Yue Zhi, Luoran Shang, Yuan Luo, Yongan Wang

**Affiliations:** ^1^ School of Clinical Pharmacy Shenyang Pharmaceutical University Shenyang China; ^2^ Academy of Military Medical Sciences Beijing China; ^3^ Pharmaceutical Sciences Laboratory Åbo Akademi University Turku Finland; ^4^ School of Medicine Southeast University Nanjing China; ^5^ Institutes of Biomedical Sciences Fudan University Shanghai China

**Keywords:** drug evaluation, hydrogel, microfluidics, neuroinflammation, organ‐on‐a‐chip

## Abstract

Neuroinflammation is a core pathological mechanism in neurodegenerative diseases. Although natural many compounds, derived from traditional Chinese medicine have shown promise in modulating neuroinflammation, conventional evaluation methods remain inefficient and fail to meet modern drug development needs. This study aimed to develop a neuroinflammation‐on‐a‐chip for efficient and accurate evaluation of the anti‐neuroinflammatory activity of such compounds. By integrating gelatin methacryloyl (GelMA) hydrogel with a microchamber array structure into a multi‐channel concentration‐gradient microfluidic chip, we constructed a functional neuroinflammation‐on‐a‐chip suitable for high‐throughput drug screening. Preliminary results demonstrated that the chip can successfully model lipopolysaccharide (LPS)‐induced neuroinflammation and test the anti‐inflammatory effects of curcumin (Cur) and resveratrol (RSV). Relative to traditional approaches, the chip offers the advantages of low sample consumption, rapid detection, and high data reliability. This study provides a novel tool for the efficient evaluation of anti‐neuroinflammatory activity of traditional Chinese medicine active compounds and offers an innovative platform for research on neuroinflammation‐related diseases.

## Key Points

- A neuroinflammation‐on‐a‐chip with a 3D biomimetic microenvironment is developed.
- It enables high‐throughput, dose‐dependent drug screening via an on‐chip gradient generator.
- The platform successfully models LPS‐induced neuroinflammatory responses.
- It validates the anti‐neuroinflammatory effects of natural compounds Curcumin and Resveratrol.

## Graphical abstract

We present a biomimetic neuroinflammation‐on‐a‐chip that models inflammatory microenvironments for efficient drug evaluation. This platform allows high‐throughput, dose‐dependent screening of natural compounds, offering a reliable tool for discovering anti‐neuroinflammatory therapeutics.

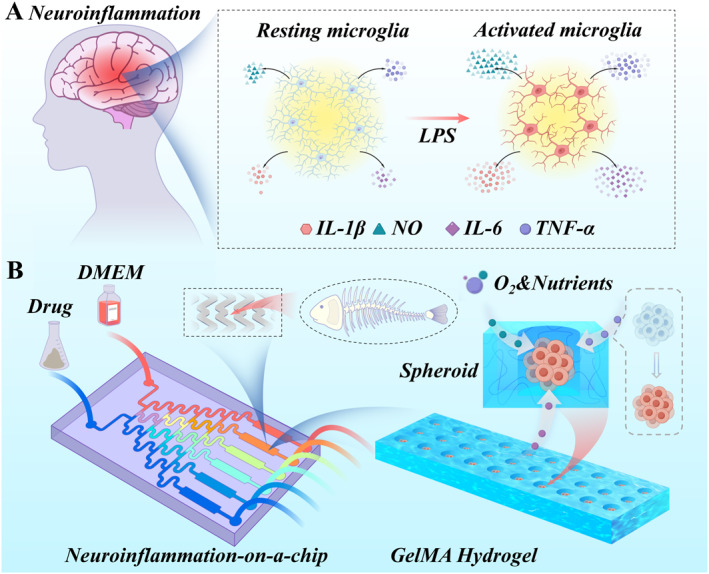

## 1 Introduction

Neuroinflammation represents an immune reaction in the central nervous system (CNS), primarily mediated by resident microglia and astrocytes. It is a key driver of the pathogenesis and progression of neurodegenerative disorders [1, 2, 3]. When the CNS is stimulated by injury, infection, or oxidative stress, microglia, as major immune effector cells, can release pro‐inflammatory factors [4, 5, 6, 7, 8, 9, 10] that exacerbate neuronal damage [11]. At present, the drugs used in clinical practice for neuroinflammation mainly include some anti‐inflammatory drugs and immunomodulators [12, 13]. Although these drugs have shown some efficacy, their clinical application is still significantly limited by side effects, and long‐term use may cause serious adverse reactions. Additionally, due to the complexity of neuroinflammation, traditional in vitro models are difficult to mimic the dynamic inflammatory microenvironment, lack physiological relevance, and are low‐throughput [14]. Therefore, the development of novel in vitro models that can mimic the dynamic processes of neuroinflammation and achieve screening of effective drugs are urgent needs for current research.

**FIGURE 1:**
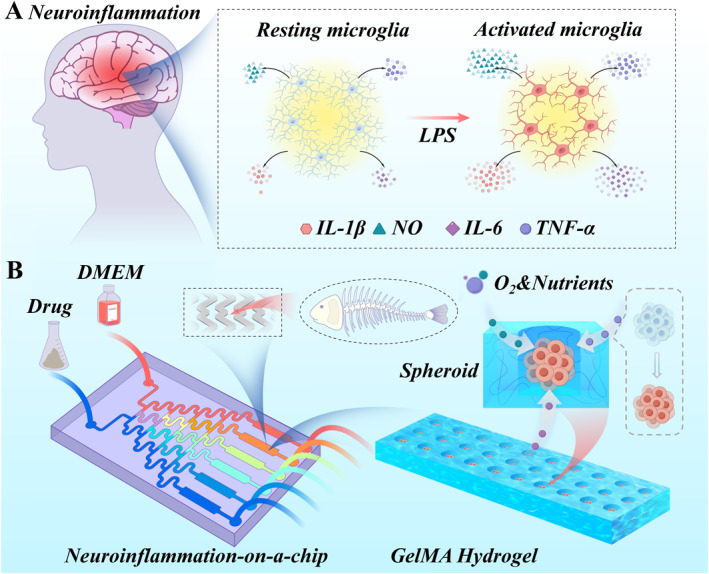
(A) LPS induces microglial activation, resulting in the release of a large number of inflammatory mediators, and triggers neuroinflammation. (B) Schematic diagram illustrates the key components and operational process of the neuroinflammation‐on‐a‐chip platform. Microglia were seeded into a microfluidic chip with multiple independent channels integrating GelMA hydrogels. The platform facilitates individualized drug screening for the treatment of neuroinflammation.

In this paper, we developed a microfluidic organ‐on‐a‐chip platform [15, 16, 17, 18, 19, 20, 21, 22] to better simulate the occurrence of neuroinflammation, and used it for high‐throughput screening of traditional Chinese medicine extracts, as shown in Figure 1. Organ‐on‐a‐chip is a miniaturized bioengineering platform that enables accurate culture and patterning of various cells, and simulates the microenvironment of human tissues/organs [23, 24, 25, 26, 27, 28, 29]. Although different kinds of organs‐on‐chips have been developed, neuroinflammation‐on‐a‐chip has been less reported. Besides, studies have proven that traditional Chinese medicine includes a variety of natural ingredients and active substances with anti‐inflammatory, antioxidant and immunomodulatory effects, which show potential in neuroinflammation treatment [30, 31, 32, 33, 34, 35, 36, 37]. Therefore, we constructed a neuroinflammation‐on‐a‐chip system to simulate neuroinflammation occurrence features and used it for screening traditional Chinese medicine active compounds.

In this study, we designed a GelMA hydrogel with a microchamber‐array structure for spheroid culture and incorporated it into a neuroinflammation‐on‐a‐chip (Figure 1). The GelMA hydrogel carrier provides an extracellular matrix‐like environment for cell seeding, and the microchamber arrays provide fixed sites for microglia culture and promote spheroid formation. The 3D spheroid culture of microglia within a tunable extracellular matrix‐like environment better mimics the in vivo cellular architecture than conventional 2D or simple 3D cultures. Even more attractive is that the herringbone microfluidic channel enhances substance exchange through turbulence, resulting in the generation of a stable drug concentration gradient. This enables dose‐dependent testing of herbal extracts such as Cur and RSV to evaluate their inhibitory effects on inflammatory cytokines. Preliminary experiments have shown that the neuroinflammation‐on‐a‐chip platform can successfully simulate LPS‐induced microglial activation and that Cur and RSV can significantly reduce the release of inflammatory cytokines. The results prove that the neuroinflammation‐on‐a‐chip provides a novel and reliable platform for screening of various active compounds derived from traditional Chinese medicine.

## 2 Results and Discussion

**FIGURE 2:**
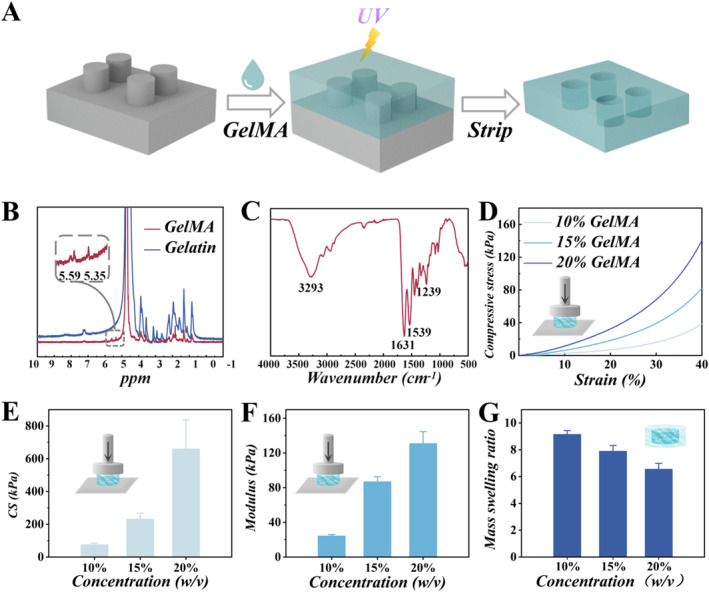
(A) Schematic of fabrication of the GelMA hydrogel with a microchamber array structure. (B) ^1^H‐NMR spectroscopy of GelMA hydrogel and gelatin. (C) FTIR spectral images of GelMA hydrogel. (D) The compressive strain‐stress curves of the different concentrations of GelMA hydrogel. (E) The compressive strength (CS) of the different concentrations of GelMA hydrogel. (F) The elastic modulus of the various concentrations of GelMA hydrogel. (G) The mass swelling ratio of GelMA hydrogel at various concentrations.

In a typical experiment, GelMA hydrogel with a microchamber array structure was fabricated via template replication method (Figure 2A). Briefly, a complete GelMA hydrogel was produced by replicating a customized PMMA template. The photograph and microscopic structures of GelMA hydrogels of different concentrations are depicted in Supporting Information S1: Figure S1A–F. Optical and SEM images show that the hydrogel was basically transparent and the average pore size decreased with the increase of the concentrations. The ^1^H‐NMR spectroscopy in Figure 2B displays the peaks at 5.35 and 5.59 ppm, which correspond to the acrylic proton of methylacrylamide. Fourier transform infrared spectroscopy (FTIR) spectra showed bands for amide A, amide I, amide II, and amide III at 3293, 1631, 1539, and 1239 cm^−1^, respectively (Figure 2C).

GelMA hydrogel samples were prepared at 10%, 15%, and 20% (w/v) concentrations to assess how concentrations affect the mechanical performance, swelling ratio, and biodegradation. The compressive strain‐stress curve, maximum compressive stress, and modulus of the GelMA hydrogel are shown in Figure 2D–F. As shown by the trend in the curves and the histogram, increasing the GelMA hydrogel concentration could improve the compressive strength and stiffness of the hydrogel. An increase in GelMA hydrogel concentration was correlated with a reduction in the swelling ratio (Figure 2G), probably because higher concentrations result in higher levels of crosslinking. Supporting Information S1: Figure S2 illustrates the degradation ratio of GelMA hydrogel in PBS. Notably, the 10% hydrogel degraded faster than those of 15% and 20% concentrations at the same observation time points, which may also be attributed to the fact that the higher concentration of GelMA hydrogel has larger crosslinking density. Considering its mechanical properties, swelling ratio, and degradation rate, the 10% GelMA hydrogel demonstrated optimal characteristics, and was chosen in subsequent experiments.

**FIGURE 3:**
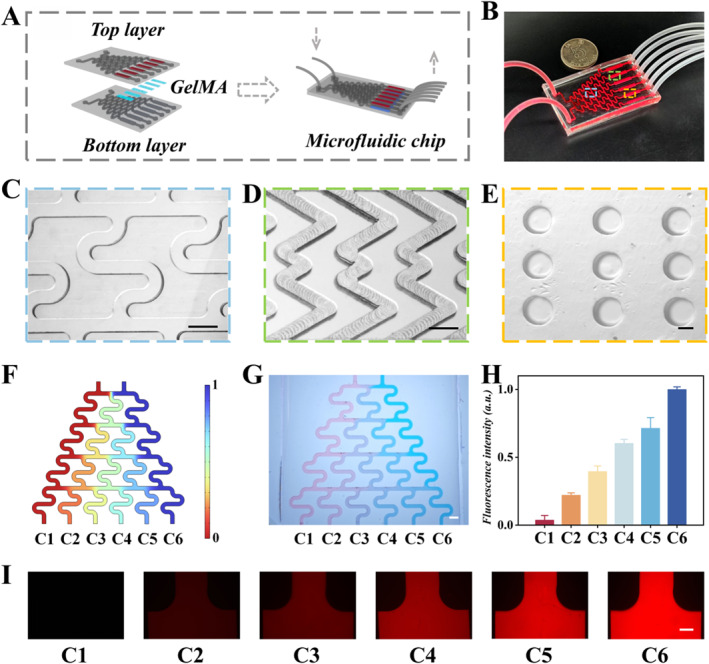
(A) Structural design diagram of the chip. (B) Image of the assembled chip. (C, D) Close‐up images showing the branching channel and herringbone structure. (E) Image of GelMA hydrogel with a microchamber‐array structure. (F) Concentration simulation results at a flow rate of 10^−8^ m/s in a microfluidic concentration gradient generator. (G) Microscopic image showing the distribution of pink and blue dyes in the microchannel of the microfluidic chip. (H) The fluorescent intensity of Rhodamine B measured at the end branches of the microfluidic channels C1 to C6 (*n* = 3). (I) Fluorescence images of Rhodamine B in the liquid within different channels on the chip mixer. The scale bars are 500 μm in (C, D), 200 μm in (E), 2 mm in (G), and 400 μm in (I).

GelMA hydrogel was integrated into a microfluidic chip. The chip featured a bottom layer of microchambers bonded with a top layer of a herringbone mixer (Figure 3A,B and Supporting Information S1: Figure S3C). The microfluidic chip comprises a concentration gradient module that employs the classic “Christmas tree” geometry to generate drug concentration gradients (Figure 3C and Supporting Information S1: Figure S3B), and a drug response module that integrates the hydrogel, enabling cells to interact independently with different drug concentrations. The top layer of the drug response module features microchannels with periodically arranged herringbone‐shaped microgrooves (Figure 3D and Supporting Information S1: Figure S3A), which facilitate enhanced mixing and substance exchange. The bottom layer of the drug response module is characterized by arrays of microchambers arranged at appropriate spacing (Figure 3E), providing a 3D microenvironment for cell culture.

The microfluidic concentration gradient generator in the chip is essential for creating precise drug gradients to determine optimal therapeutic concentrations. Numerical simulations were conducted to display a clear drug concentration gradient from channel 1 to channel 6 (C1‐C6) (Figure 3F). Pink and blue dyes were pumped into the two inlets (Figure 3G) resulting in a significant gradient. When Rhodamine B and ultrapure water were pumped, distinct color differences of the six outlet solutions are shown in Figure 3H,I. This integrated gradient system enables the chip to perform parallel testing of multiple concentration gradients in a single run, demonstrating its advantage over conventional methods in terms of sample consumption, operational time, and data consistency.

**FIGURE 4:**
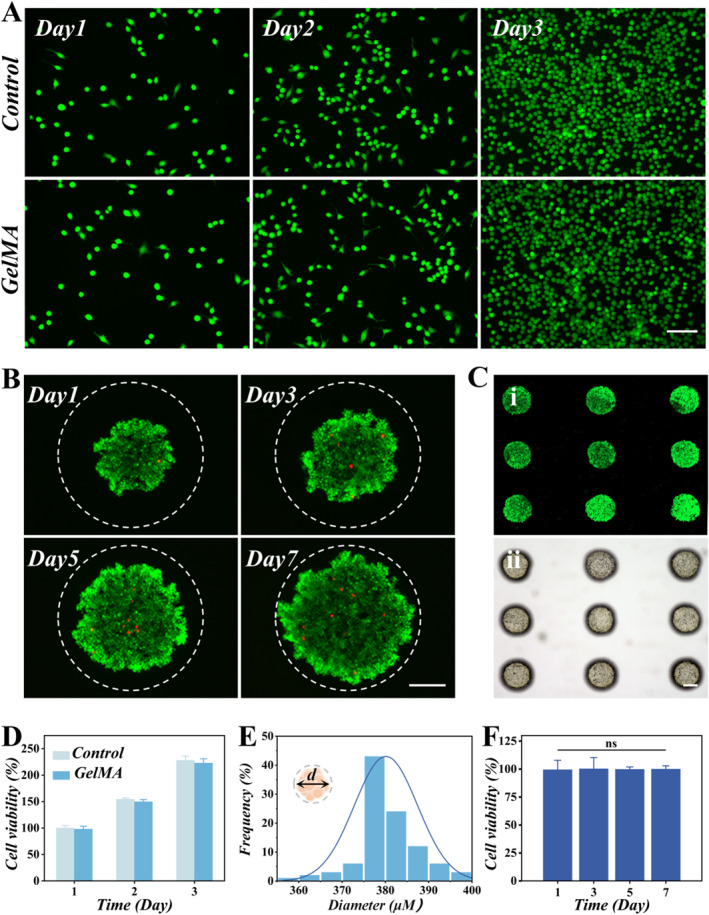
(A) Live staining on BV2 cells for 1, 2, and 3 days of culture. (B) Live/dead staining of BV2 cell spheroids in the microchambers at 1, 3, 5, and 7 days. (C) Fluorescence microscopic (i) and microscopic images (ii) of spheroids growing in the chip. (D) BV2 microglial viability in Control and GelMA groups. (E) The size distribution of spheroids on day 7 (*n* = 100). (F) Quantitative analysis of BV2 cell spheroid viability on culture days 1, 3, 5, and 7 (*n* = 10). Scale bars are 50 μm in (A), 100 μm in (B), and 200 μm in (C).

After conducting functional tests on the chip, we evaluated the biocompatibility of the GelMA hydrogel. The corresponding hydrogel was soaked in the cell culture medium to obtain the leachate for subsequent experiment. As illustrated in Figure 4A,D, BV2 microglia demonstrated good biocompatibility in GelMA hydrogel leachate. We then seeded BV2 microglia into the microchambers of the microfluidic chip and cultured them for 7 days, systematically monitoring their proliferation and morphological behavior. The GelMA hydrogel, coated with 0.75% Polyvinyl alcohol (PVA) solution, allowed the cells to spontaneously organize into small clusters within the microchambers, eventually forming cell spheroids (Figure 4C). In addition, cell proliferation and viability were also evaluated throughout the experimental period by live/dead staining and subsequent quantitative analysis (Figure 4B,F). We measured the spheroid size on day 7 of culture, and the results showed that the spheroids cultured in GelMA hydrogel microchambers exhibited uniform size (Figure 4E). The cells exhibited good viability over the 7‐day culture period compared to those cultured in a plate. The culture medium was maintained in a dynamic flow state, which ensures a continuous supply of O_2_ and nutrients to the interior of the spheroids, and the metabolic waste can be carried away by the flowing culture medium. Thus, the hydrogel‐integrated chip provides a good living environment for spheroids.

**FIGURE 5:**
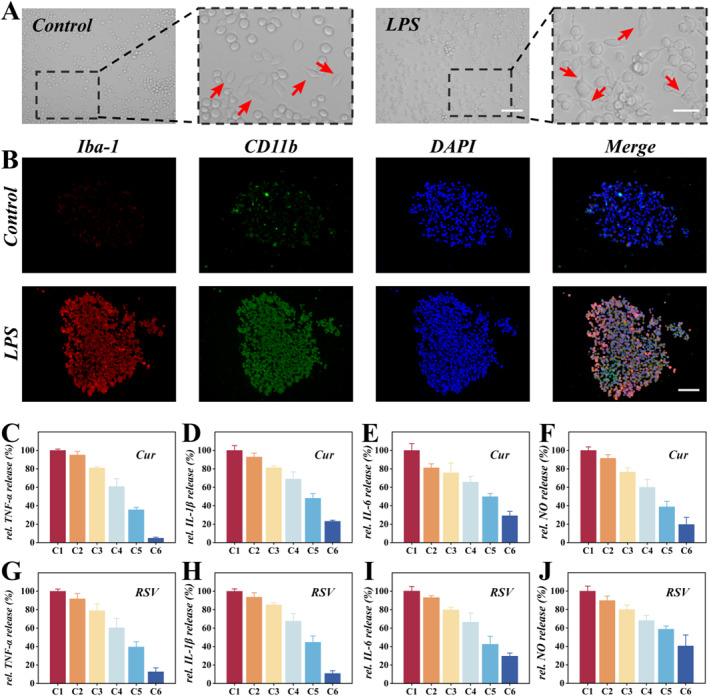
(A) Morphological changes in BV2 microglia with or without LPS stimulation. (B) Immunofluorescence staining for Iba‐1 and CD11b in BV2 spheroids. (C–F) Drug screening results for Cur. (G–J) Drug screening results for RSV. The scale bars are 100 μm in (A, B), 50 μm in the enlarged image in (A).

It is reported that LPS activates microglia and induces an inflammatory response, and the TLR4 receptor can mediate this process. The release of nitric oxide (NO) in cells stimulated by different concentrations of LPS was detected using an NO test kit. Supporting Information S1: Figure S4A illustrated that the expression of NO in cells increased with the increasing concentration of LPS. However, Supporting Information S1: Figure S4B indicates that higher concentrations of LPS can cause significant cellular damage. Therefore, a concentration of 1 μg/mL of LPS was selected to act on cells for 24 h to construct an in vitro model of neuroinflammation. We characterized the morphology of microglia 24 h after LPS treatment and found that these cells exhibited an ameba‐like morphology (indicated by red arrows) (Figure 5A and Supporting Information S1: Figure S5). Furthermore, we measured the release of inflammatory factors and NO from cell spheroid induced for 24 h with or without 1 μg/mL LPS (Supporting Information S1: Figure S6). These findings suggest that LPS stimulation significantly induces BV2 microglia activation and promotes their release of inflammatory mediators.

In order to verify the successful construction of the neuroinflammation model, we performed immunofluorescence staining on spheroids to evaluate the CD11b and Iba‐1 expression. As depicted in Figure 5B, LPS‐induced upregulation of CD11b and Iba‐1 expression in spheroids was observed, suggesting that the microglia have been activated. To achieve high‐throughput drug screening, we first evaluated the drug safety of Cur and RSV. Both compounds exhibit poor solubility in aqueous culture medium. In this study, Cur and RSV were first dissolved in DMSO and subsequently diluted in culture medium to a final DMSO concentration of ≤ 0.1% to ensure solubility and maintain biosafety (Supporting Information S1: Figure S7). To assess the anti‐inflammatory effects of Cur and RSV against LPS‐treated BV2 microglia, we infused culture medium through one inlet of the chip and separately delivered Cur and RSV through the other inlet. After 1 h of incubation, BV2 cells were induced with LPS (1 μg/mL) followed by collection of culture supernatant from six outlets for quantification of NO and inflammatory factors. The quantitative data indicated that with the increase in Cur or RSV concentration, the NO and inflammatory factors continued to decrease (Figure 5C–J). These results demonstrate the effectiveness of the neuroinflammation‐on‐a‐chip constructed by integrating the GelMA hydrogel with a microchamber array structure and a microfluidic chip in drug screening. This functional versatility stems from the platform's modular design and broad material compatibility, which allow it to process not only natural compounds but also a variety of synthetic molecules and biological agents. Furthermore, by integrating relevant cell types or pathological stimulation, the platform can be adapted to model a wider range of diseases.

## 3 Conclusion

In conclusion, we designed a neuroinflammation‐on‐a‐chip by combining GelMA hydrogel with a microfluidic chip featuring a special herringbone structure for microglia culture and drug screening. Within the chip, microchamber arrays allow the cells to form cell spheroids and maintain a high survival rate. In addition, by combining with a multi‐channel concentration gradient generator, the functional neuroinflammation‐on‐a‐chip allows for high‐throughput and high‐precision drug screening. While the current platform focuses on acute inflammation modeling, it provides a flexible foundation for future integration of chronic disease models. Therefore, the designed neuroinflammation‐on‐a‐chip is expected to be an ideal platform for screening drugs for the treatment of neuroinflammation and various diseases.

## 4 Methods

### 4.1 Materials

GelMA hydrogel was self‐synthesized from gelatin (Sigma). Polydimethylsiloxane (PDMS) was purchased from Dow Corning. DAPI and NO test kits were obtained from Beyotime. LPS and Triton X‐100 were acquired from Sigma. PVA was obtained from Aladdin. Phalloidin was purchased from Thermofisher. paraformaldehyde was purchased from biosharp. 2‐hydroxy‐2‐methylpropiophenone (HMPP), Cur, RSV, Bovine Serum Albumin (BSA), and methacrylic anhydride were acquired from Macklin. Calcein‐AM/PI was purchased from Molecular Probes. BV2 (CL‐0493) was acquired from Pricella Biotechnology. FBS, DMEM, and PBS (pH 7.4) were obtained from Gibco. IL‐1β and IL‐6 ELISA kits were acquired from Dogesce. TNF‐α ELISA kit was obtained from Vicky. Cell Counting Kit‐8 (CCK8) was acquired from MedChemExpress (MCE).

### 4.2 Preparation of GelMA Hydrogel

GelMA Hydrogels of different concentrations were fabricated by dissolving lyophilized GelMA in PBS containing HMPP photoinitiator. The solutions were then photopolymerized under ultraviolet light for 30 s. Images of the hydrogel samples were captured to characterize their appearance.

### 4.3 Physiochemical Characterization of GelMA Hydrogel

The ^1^H NMR spectra of GelMA hydrogel and gelatin were evaluated using the NMR spectrometer (QUANTUM‐1–400 MHz). FT‐IR testing of GelMA hydrogel was carried out using a spectrometer (Tensor II). GelMA hydrogel samples of different concentrations were prepared, freeze‐dried, sprayed with gold, and their microscopic morphologies were characterized using a scanning electron microscope (SEM, SU8010).

The compressive mechanical properties of 10%, 15%, and 20% GelMA hydrogels were evaluated using an electronic universal testing material machine (5944, Instron). The crosshead speed was set at 2 mm/min. Cylindrical hydrogels (diameter: 6.5 mm, height: 5 mm, *n* = 3) were prepared and tested. We plotted the compressive strain‐stress curves, recorded and calculated the modulus and maximum compressive stress. For each hydrogel's concentration, we tested three samples to obtain the mean and standard deviation.

We fabricated GelMA hydrogels (diameter: 7 mm, height: 4 mm, *n* = 3) for swelling ratio analysis. The samples were immersed in PBS until they reached equilibrium swelling. After immersion, we gently blotted the samples with Kimwipes to remove excess liquid, recorded the wet weight, froze them at −80°C for 2 h, lyophilized them overnight, and then measured their dry weight. The swelling ratio was quantified by (wet mass ‐ dry mass)/dry mass.

GelMA hydrogel samples at various concentrations were prepared into cylindrical shapes (diameter: 7 mm, height: 4 mm, *n* = 3). The samples were initially immersed in 2 mL of sterile PBS at 37°C to reach swelling equilibrium. Subsequently, Samples were collected at 0, 2, 4, 6, 8, 10, 12, and 14 days, and their masses were recorded as *W*d (with *W*d_0_ representing the initial mass and *W*d_n_ representing the mass at each subsequent time point). The degradation ratio was determined using the formula: (*W*d_0_ − *W*d_n_)/*W*d_0_ × 100%.

### 4.4 Construction of Microfluidic Chips

The PDMS layers of the chip were constructed by replicating the molds. Firstly, the mold was coated with a PDMS (10:1 base to curing agent, w/w). Then, the air bubbles are evacuated, and the mixture was left in the oven overnight. Next, the chip layers were peeled from the mold using tweezers. Immediately after treatment with oxygen plasma, two PDMS layers were clamped in a vacuum environment for 1 h for bonding.

### 4.5 In Vitro Biocompatibility Characterization

In the experimental group, GelMA hydrogel was immersed in cell culture medium for 24 h, followed by filtering to remove impurities to obtain the leachate. The control group used cell culture medium. The toxic effects of GelMA hydrogel on BV2 cells were detected by using leachate and ordinary medium for the culture of BV2 cells, respectively. In a typical procedure, BV2 microglia were seeded in 2D plates and cultured for 1–3 days. Live staining experiments were performed on days 1, 2, and 3 according to the manufacturer's manual. Cell viability in each group was assessed using the CCK8 assay. Three samples were set up per set.

### 4.6 Characterization of BV2 Cell Morphology

BV2 microglia were treated with LPS and subsequently stained for F‐actin and nuclei to assess morphological changes. Briefly, 4% paraformaldehyde was used to fix the samples, permeabilized with Triton X‐100 (0.1%) and stained with phalloidin‐iFluor 488 (1:400) for 30 min and DAPI for 10 min. Confocal microscopy captured the fluorescence images. (Evident/FV3000).

### 4.7 Culture of Cell Spheroids

GelMA hydrogel with a microchamber array structure was sterilized under UV light for 6 h, sprayed with 0.75% PVA solution, and then assembled into the chip. The BV2 cell suspension was pumped into the chip and cultured to form BV2 spheroids. The size of the BV2 spheroids was determined by measuring them under a microscope on days 1, 3, 5, and 7. At each time point, the spheroids were incubated with Calcein‐AM/PI (1 μL/mL) for 20 min at 37°C and imaged with a fully automated fluorescence scanning imaging system (BZ‐X800LE). The spheroids were incubated with CCK8 (100 μL/mL) for 2 h, and measured the absorbance. When the cells grew close to the mold size, they were imaged using an LSM980 laser scanning confocal microscope.

### 4.8 Immunofluorescence Staining

4% paraformaldehyde was used to fix the samples and permeabilized with Triton X‐100 (0.1%). Following a 30‐min block in 3% BSA, the spheroids were incubated with Iba‐1 (1:500) and CD11b (1:400) specific primary antibodies overnight at 4°C. They were then incubated with the corresponding fluorescent secondary antibody for 50 min followed by DAPI for 10 min.

### 4.9 Drug Screening

We cultured the spheroids for 7 days, treated them with drugs for 1 h, and subsequently stimulated them with LPS. We collected the liquid from six outlets and clarified it by centrifugation (3000 rpm, 5 min) to eliminate cell debris. We then added the liquid to a 96‐well plate, and added the Griess Reagent. After a 15‐min incubation, absorbance was read at 540 nm. NO level was quantified against a sodium nitrite‐derived standard curve (0–100 μM). Finally, according to the instructions, inflammatory factor levels were determined by the ELISA kit.

### 4.10 Characterization

Images of the chip and hydrogel were taken using a microscope. Microscopic images of BV2 cells were captured using a fully automated fluorescence scanning imaging system (BZ‐X800LE). Fluorescence images of different concentration gradients in the chip were captured using an inverted microscope (Axio Vert. A1, ZEISS).

### 4.11 Statistical Analysis

All results were obtained as mean ± SD from no fewer than three independent replicates and are presented. Statistical significance was assessed by *t*‐test. Significance was defined as follows: ns, not significant; *p < 0.05; **p < 0.01; ***p < 0.001.

## Author Contributions

Y.W., Y.L., and L.S. conceived the idea and designed the experiment. X.W. and X.L. conducted experiments and data analysis. X.W. and Y.Z. wrote the manuscript.

## Ethics Statement

The authors have nothing to report.

## Conflicts of Interest

Luoran Shang is an executive editor for *Smart Medicine* and was not involved in the editorial review or the decision to publish this article. All authors declare that there are no competing interests.

## Supporting information

Supporting Information S1

## Data Availability

The data that support the findings of this study are available on request from the corresponding author. The data are not publicly available due to privacy or ethical restrictions.
