# Supplementary material for: Constructing Neuroinflammation‐On‐A‐Chip for Traditional Chinese Medicine Extracts Evaluation
Source: Smart Med. 2026 Mar 19;5(2):e70032. doi: 10.1002/smmd.70032 (PMC13131082; doi:10.1002/smmd.70032)
Supplement: Supplementary file 1 — Supporting Information S1 [file SMMD-5-e70032-s001.docx]

**Supplementary**

**Constructing neuroinflammation-on-a-chip for traditional Chinese medicine extracts evaluation**

Xirui Wang^1&2^, Xiang Lin^3^, Yue Zhi^4^, Luoran Shang^5^*, Yuan Luo^2^*, Yongan Wang^1&2^*

^1^ School of Clinical Pharmacy, Shenyang Pharmaceutical University, Shenyang 110016, China

^2^ Academy of Military Medical Sciences, Beijing 100850, China

^3^ Pharmaceutical Sciences Laboratory, Åbo Akademi University, Turku 20520, Finland

^4^ School of Medicine, Southeast University, Nanjing 210009, China

^5^ Institutes of Biomedical Sciences, Fudan University, Shanghai 200032, China

Email: luoranshang@fudan.edu.cn (L. Shang), [luoyuan2006@163.com (Y. Luo),](mailto:luoyuan2006@163.com,) [yonganw@126.com](mailto:yonganw@126.com) (Y. Wang)


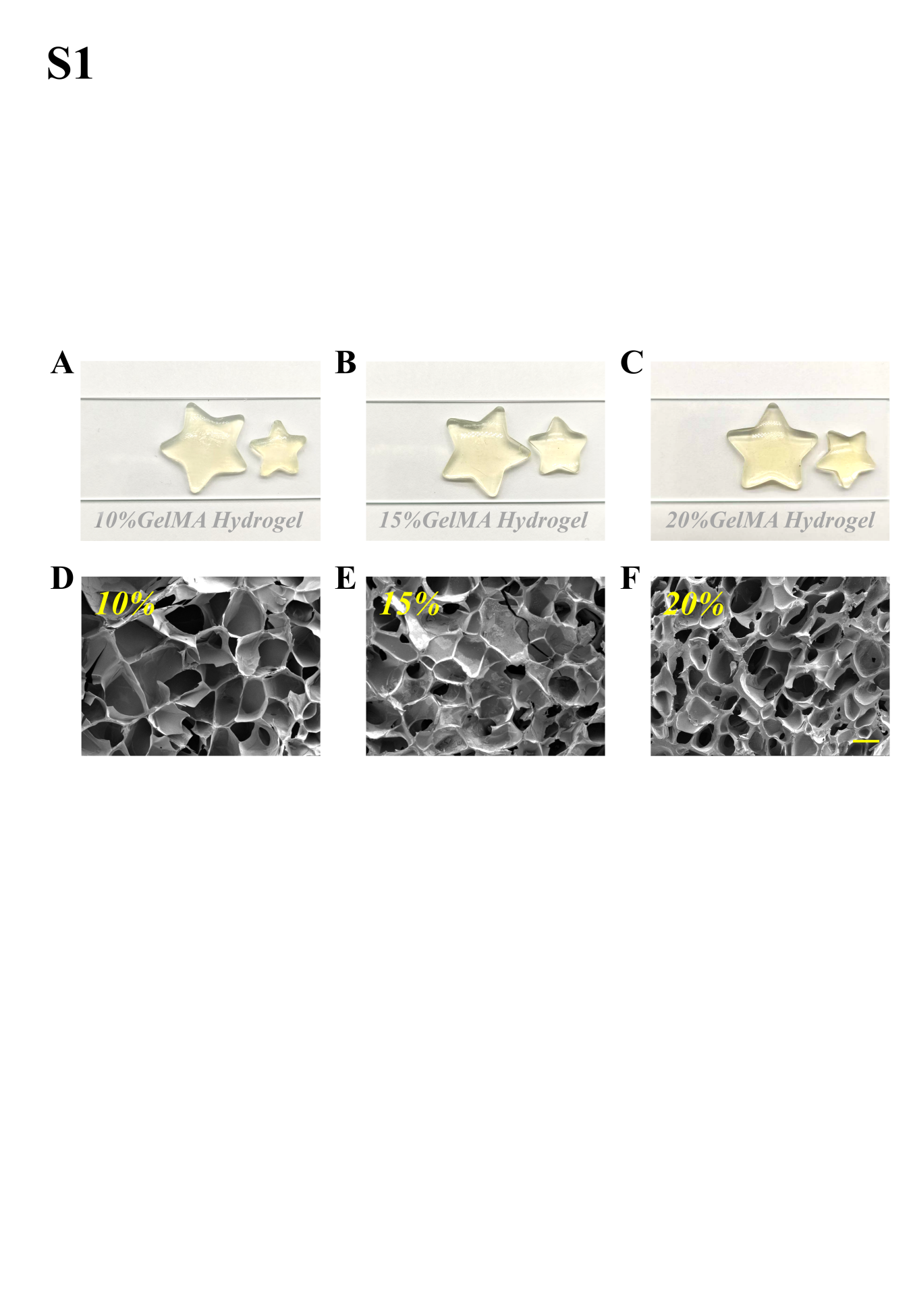


**Figure S1.** A-C) Photographs of GelMA hydrogels of 10%, 15%, and 20% concentrations. D-F) SEM images showing the porous structure of GelMA hydrogel at concentrations of 10%, 15%, and 20%, after freezing drying. The scale bar is 200 μm.


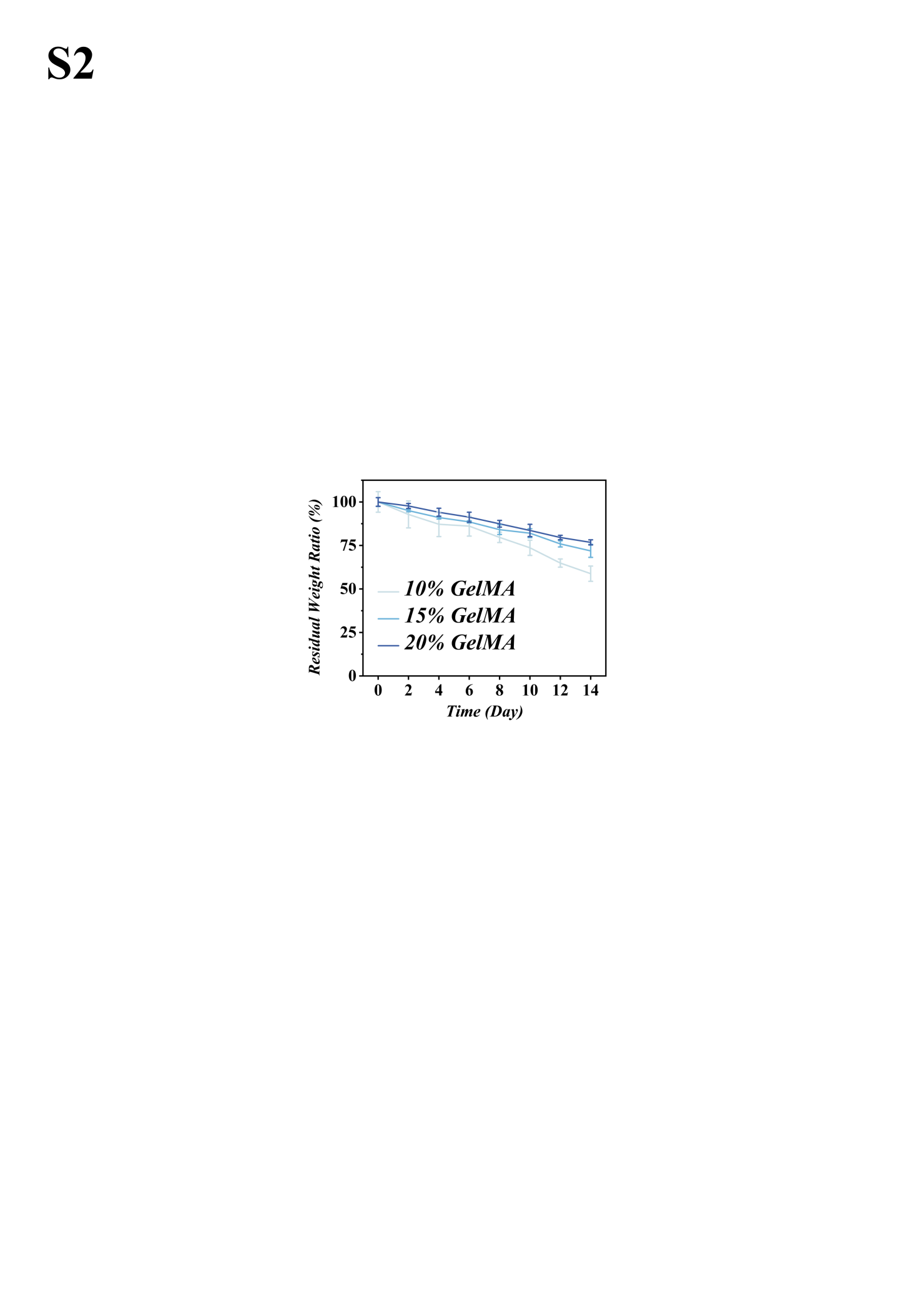


**Figure S2.** Biodegradation of GelMA hydrogel in PBS.


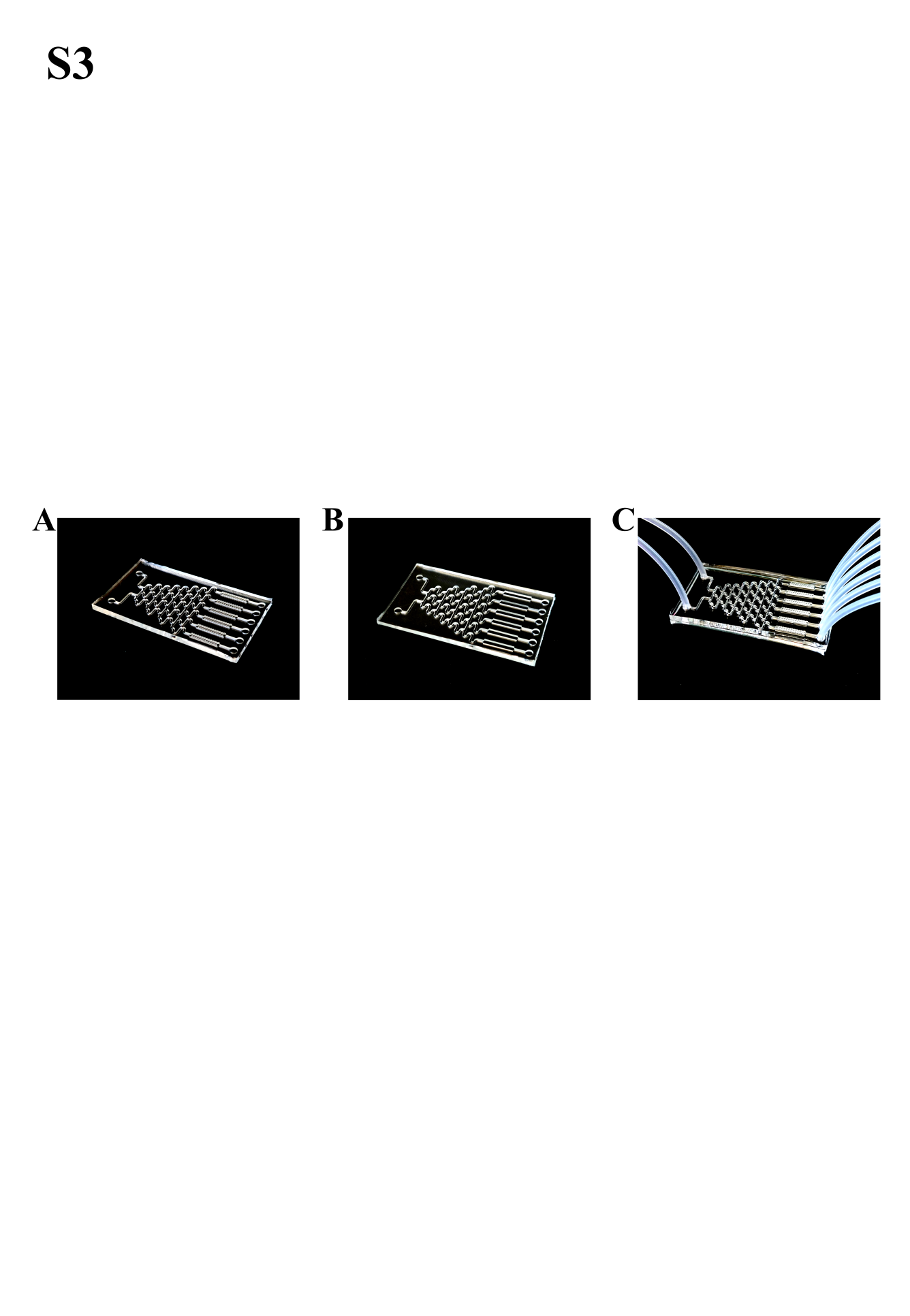


**Figure S3.** A-B) Chip top and bottom layer images. C) Photograph of the assembled chip.


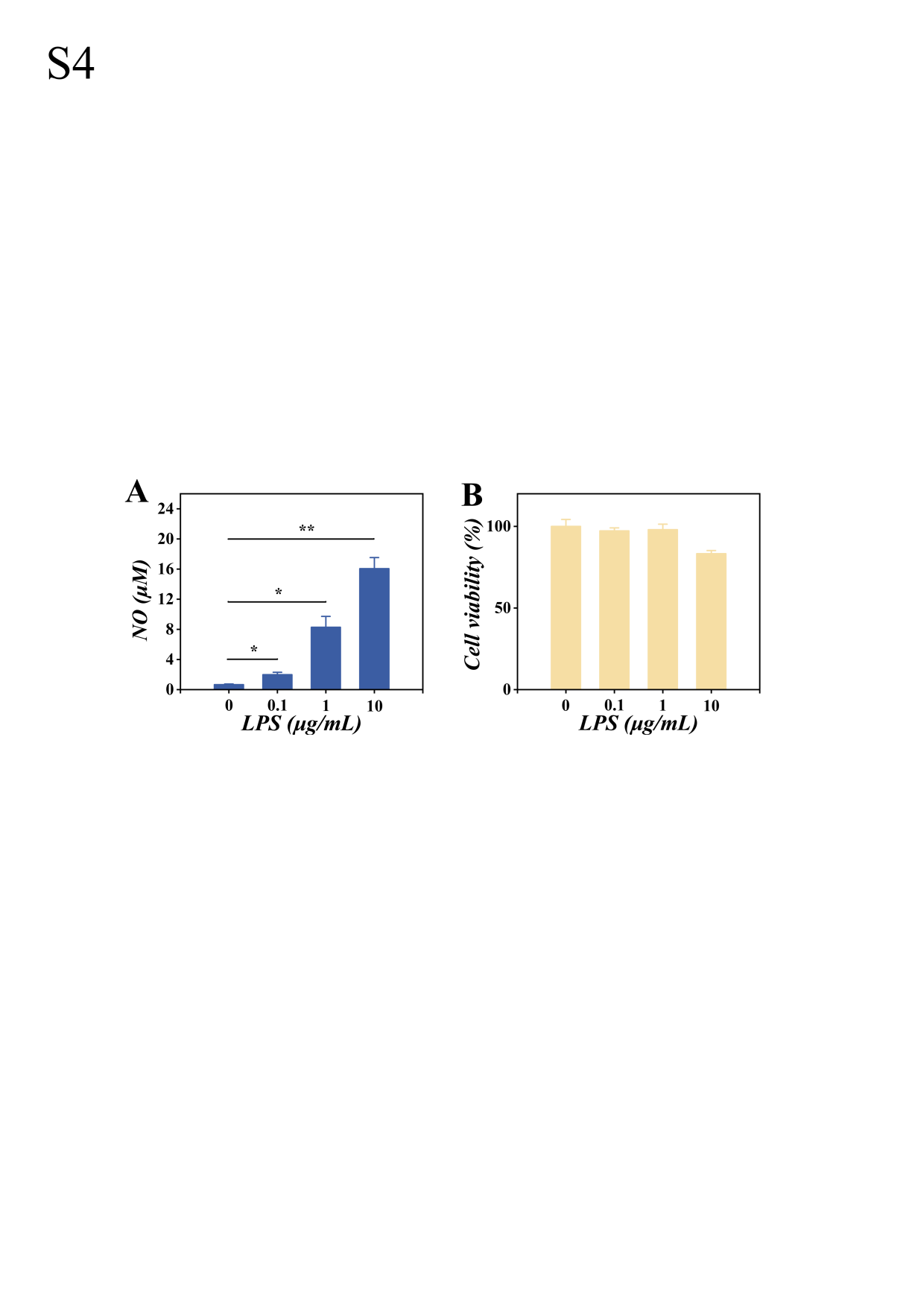


**Figure S4.** A) Effect of LPS on NO synthesis in BV2 microglia. B) Effect of LPS treatment on BV2 viability.


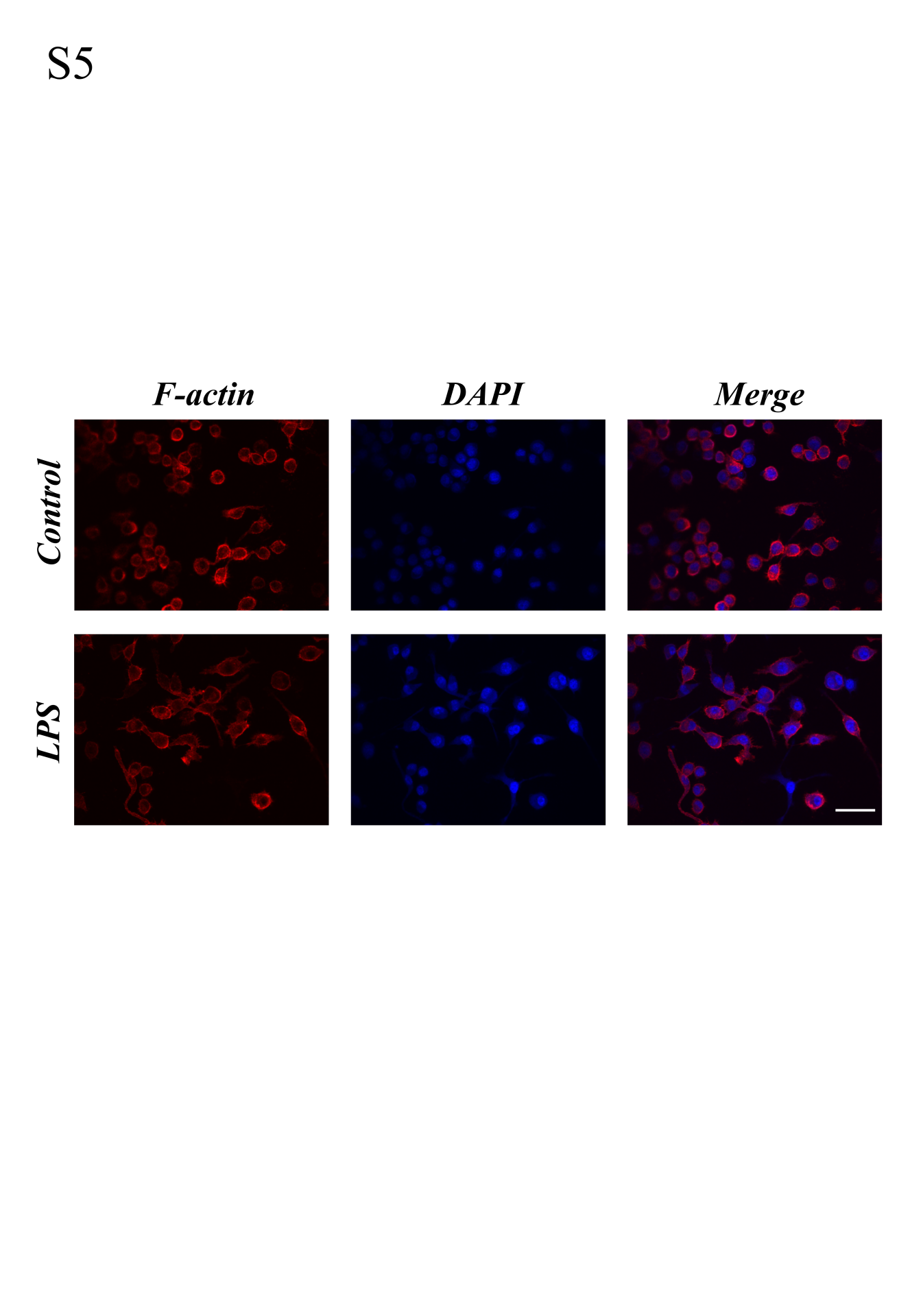


**Figure S5.** Morphological changes in the resting state (Control) of BV2 microglia and after LPS stimulation. The scale bar is 100 μm.


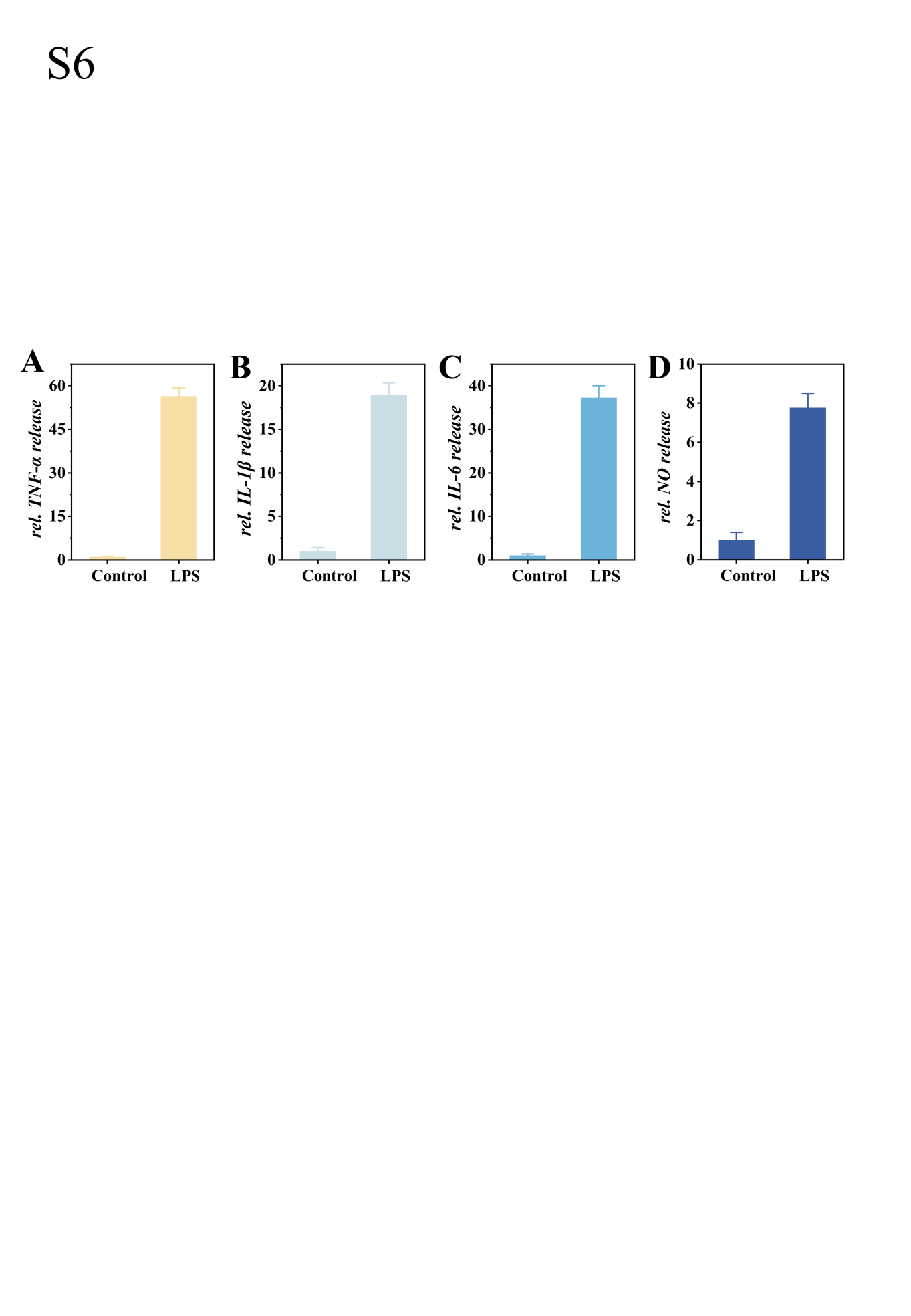


**Figure S6.** A-D) Release of TNF-α, IL-1β, IL-6, and NO from cell spheroid induced with or without LPS for 24 h.


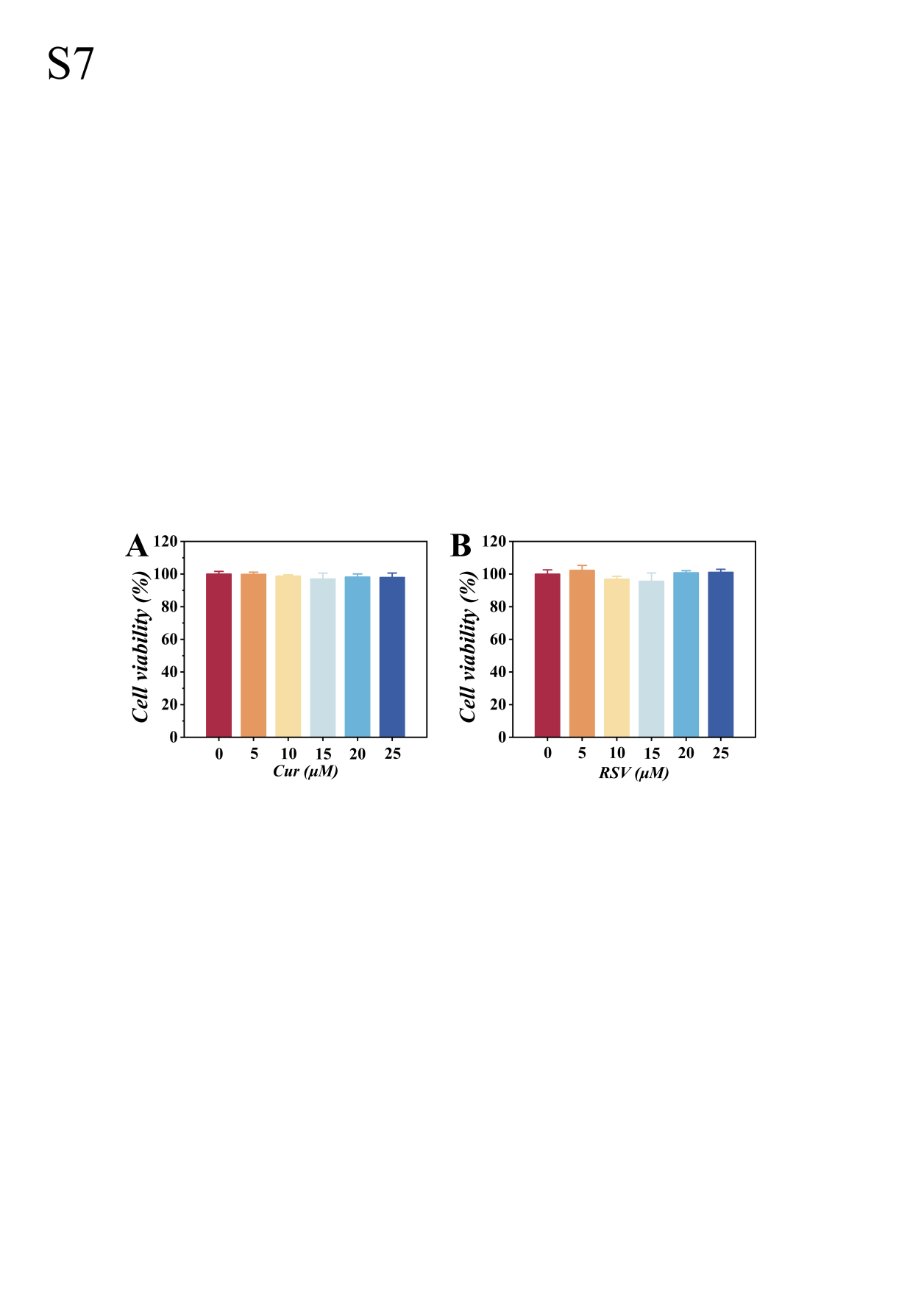


**Figure S7.** A) Viability of BV2 microglia treated with Cur. Concentrations C1 to C6 were 0, 5, 10, 15, 20, and 25 μM, respectively. B) Viability of BV2 microglia treated with RSV. Concentrations C1 to C6 were 0, 5, 10, 15, 20, and 25 μM, respectively.
